# Arrested Cells/Cellular Debris Expelled from Blastocysts Is Self-Correction Phenomenon During Early Embryonic Development

**DOI:** 10.1007/s43032-022-01159-8

**Published:** 2023-01-10

**Authors:** Xiaoxia Wang, Jing Zhao, Zhongyuan Yao, Qiuping Xia, Tianli Chang, Jun Zeng, Jiaqi Liu, Yanping Li, Huimin Zhu

**Affiliations:** 1 Department of Reproductive Center, Xiangya Hospital, Central South University, Changsha, 410078 Hunan China; 2 Clinical Research Center for Women’s Reproductive Health in Hunan Province, Xiangya Hospital, Central South University, Changsha, 410078 Hunan China; 3 Center for Medical Genetics & Hunan Key Laboratory of Medical Genetics, School of Life Sciences, Central South University, Changsha, 410000 Hunan China; 4 Yikon Genomics Company, Ltd, No.218, Xinghu Street, Suzhou, 215000 China; 5 The Fourth Affiliated Hospital, Zhejiang University School of Medicine, Yiwu, 322000 Zhejiang China

**Keywords:** Embryonic self-correction, Arrested cells/cellular debris, Trophectoderm biopsy, Aneuploidy, Mosaicism

## Abstract

Arrested cells/ cellular debris is component left in the zona pellucida after blastocyst hatching. To identify whether expelling arrested cells/cellular debris from blastocysts is a process of human embryo self-correction by eliminating abnormal cells, 21 pairs of trophectoderm (TE) biopsies and the corresponding arrested cells/cellular debris expelled from the blastocysts from July to December 2020 were collected and analyzed using next-generation sequencing (NGS). Then, the NGS results of TE biopsies and the corresponding arrested cells/cellular debris were compared. We identified that 47.6% of blastocysts (10/21) were aneuploidies and mosaicism. A total of 18 groups of arrested cells/cellular debris (85.7%) expelled from blastocysts were abnormal, including nine aneuploid embryos and nine euploid embryos. In the arrested cells/cellular debris, all the chromosomes were affected. In conclusion, mosaicism and aneuploidies are common features of early embryonic development, and the arrested cells/cellular debris expelled from blastocysts provides evidence of early embryonic self-correction.

## Introduction

Mosaic and aneuploid embryos in human are prevalent throughout pre- and post-implantation development [1, 2]. Chromosomal abnormalities in early embryos result from errors during gamete meiosis (most commonly being seen in uniform aneuploidies) and errors during blastomere mitosis (mainly resulting in mosaicism) [3, 4]. When chromosomes fail to separate or separate prematurely into sister chromatids during gamete meiosis, both conditions can result in aneuploidies and then occur in all embryonic cells. That is because when errors occur during meiosis, aneuploid cells are prevalent in human embryos accompanied with cell division after fertilization. Nonetheless, if chromosome separation errors occur in an embryonic cell during mitosis, a mosaic embryo containing diploid and aneuploid cells can be formed consequently [3, 5]. Research has confirmed that the initial stages of human embryonic development are characterized by rapid cell division, which may lead to chromosomal instability resulting in either chromosomal mosaicism or rearrangement [2, 3]. Besides, chromosomal abnormalities in embryos are related to maternal age, embryo morphology, and development speed [6, 7].

Previous studies relied on animal models to investigate chromosomal abnormalities in embryos [8, 9]. However, those models are extremely difficult to provide first-hand evidence for self-correction mechanisms in human embryos. The advancement of in vitro fertilization/intracytoplasmic sperm injection (IVF/ICSI) and cytogenetic techniques for preimplantation genetic testing (PGT) further enhance our understanding of the chromosomal abnormalities in human gametes and preimplantation embryos. Even more intriguing is that several studies reported that human mosaic embryos were capable of producing healthy euploid babies [10–12]. It seems that embryos have self-correction ability to rectify chromosomal errors. Some other studies showed that although during later stages of development, chromosomal abnormalities and mosaicism were still found in blastocysts, data indicated a reduced proportion of aneuploid cells, and even 9.7–40% of aneuploid embryos transformed into euploidies by undergoing complete self-correction [13, 14]. Santos et al. also revealed a marked reduction in the number of abnormal cells in 4, 5, and 8-day embryos over time [15]. Similarly, blastocysts diagnosed with mosaicism when being cultured to day 12 also have been noticed to show euploidy profiles in both ICM and TE-derived lineages, as observed by Popovic et al. [16]. Moreover, euploid human embryonic stem cells can be derived from abnormal embryos, implying that aneuploid cells may be depleted in the presence of euploid cell competition [17]. Thus, a possible hypothesis of a reduced rate of aneuploidies during the blastocyst stage of embryonic development is that an embryo can eliminate its aneuploid cells [18, 19].

**Fig. 1:**
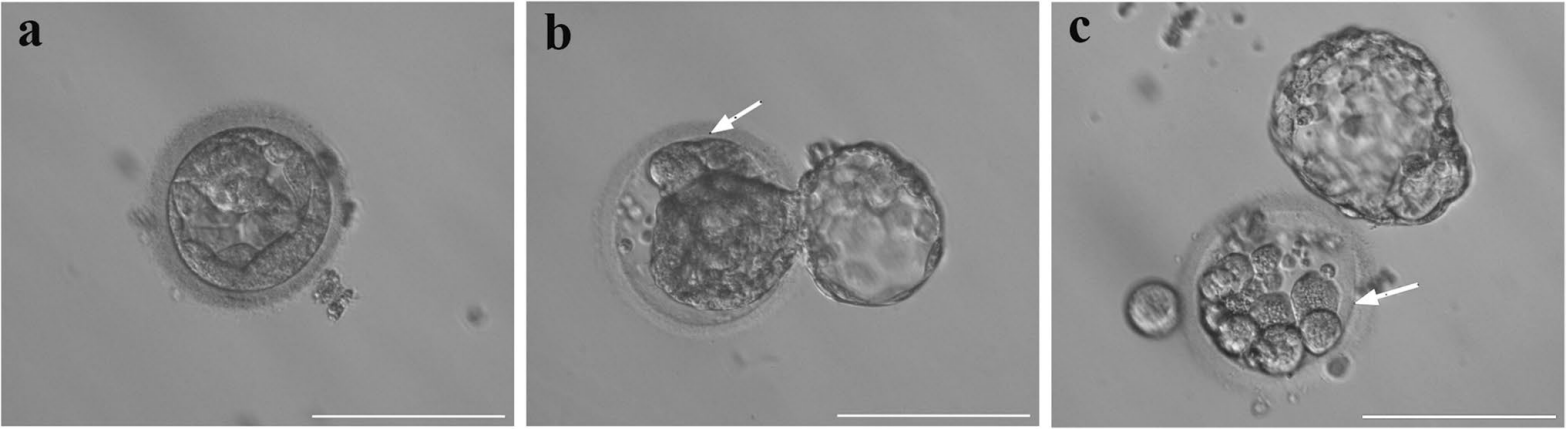
Blastocysts with or without expelled arrested cells/cellular debris. **a** A blastocyst without hatching. **b**, **c** Hatching blastocysts with arrested cells/cellular debris left in zona pellucida. White arrows indicated arrested cells/cellular debris

In our daily clinical work, we observed that some cells were eliminated from the blastocysts and left in the zona pellucida after blastocyst hatching (Fig. 1). Because the component of them was unknown, we called them arrested cells/cellular debris. Based on the aforementioned findings, we have hypothesized that this might represent a “correction” mechanism that rescues embryos from mosaicism or aneuploidy by eliminating abnormal cells, and it typically occurs after abnormal cleavage. In the present study, we detected the arrested cells/cellular debris isolated from blastocysts and performed a consistent comparison with the corresponding TE biopsy results to verify the hypothesis of the self-correction mechanism during early embryonic development.

## Materials and Methods

### Material Source and Ethical permission

In this study, 21 embryos with arrested cells/cellular debris were obtained from 19 patients who accepted PGT at the Department of Reproductive Medicine, Xiangya Hospital, Central South University from July 1st to December 31st, 2020. The indication of PGT included monogenetic disorders, chromosomal rearrangement, repeated implant failure, and unexplained repeated pregnancy loss. The study was approved by the Institutional Review Board of Xiangya Hospital, Central South University. All patients signed informed consent forms and agreed to participate the study.

### Embryo Culture and Sampling

Conventional controlled ovarian hyperstimulation was used before ovum pick-up. Then, the collected oocytes were fertilized in vitro by intracytoplasmic sperm injection (ICSI). On days 1, 2, and 3 after fertilization, the embryos were morphologically evaluated and recorded according to Istanbul consensus [20]. On the days 5 or 6, the blastocyst morphology was examined under an inverted microscope to differentiate between TE and ICM. Blastocysts were graded using the Gardner grading system [21], and those with scores of 3BC, 3CB, or better were hatched by using assisted laser hatching for zona pellucida opening. Then, after 4–6 h of blastocyst incubation, 5–8 TE cells were separated using the microlaser-blunt dissection method and aspirated into a biopsy pipette. Besides, a biopsy of the arrested cells/cellular debris remaining in the zona pellucida was also collected and put into a separate polymerase chain reaction tube. Following biopsies, all blastocysts were vitrified using vitrification protocol for personalized embryo transfer in the next cycle after knowing the PGT results.

### Sample Processing and Testing

All testing was conducted by Yikon Genomics according to previously described methods [22–24]. Briefly, all TE samples and arrested cells/cellular debris were separately amplified by the multiple annealing and looping-based amplification cycles (MALBAC) single-cell whole-genome amplification kit (Yikon, China). The amplified products were purified using CMPure magnetic beads, and electrophoresis was used to ensure quality control. The target fragmentation was realized using the Covaris M220 DNA Shearing instrument for genomic library construction. NGS testing was carried out using an Illumina HiSeq 2500 system after purification and library quality testing. Sequencing yielded no less than 2 million reads for a single sample. Approximately 20–80% of abnormal cells were classified as mosaicism. One less than 20% of mosaic aneuploid cells was reported as euploidy, and one more than 80% was reported as aneuploidy.

### NGS Protocol Validation

Before the initiation of our study, the NGS protocol was validated for accuracy as reported previously [25]. Briefly, Preimplantation Genetic Testing-Aneuploidy kits (semiconductor sequencing) for library construction were tested for accuracy periodically. The total library construction failure rate should not be >3%, the valid data should not be <1Mb, and the genomic coverage should not be <4%. Only if the tested PGT-A met the quality control, they could be used for clinical sample sequencing.

## Results

The present study recruited 19 patients (30.59 ± 5.38 years for average female age) who underwent PGT and analyzed a total of 21 pairs of TE biopsies and the arrested cells/cellular debris from their embryos. Couples were from three different groups, including preimplantation genetic testing for chromosomal structural rearrangements (PGT-SR), preimplantation genetic testing for monogenic/single gene defects (PGT-M), and preimplantation genetic testing for aneuploidy (PGT-A). Their ages, embryo morphological grading, and NGS results of their TE biopsies and the corresponding arrested cells/cellular debris are shown in Table 1. In the 21 pairs of TE biopsies and arrested cells/cellular debris, seven pairs were from six couples (27.33 ± 2.69 years for average female age) who accepted PGT-SR, three pairs were from three couples (29.33 ± 1.70 years for average female age) who accepted PGT-M, as well as 11 pairs were from ten couples (32.90 ± 5.89 years for average female age) who accepted PGT-A because of repeated implantation failures or recurrent pregnancy loss.

**Table 1:**
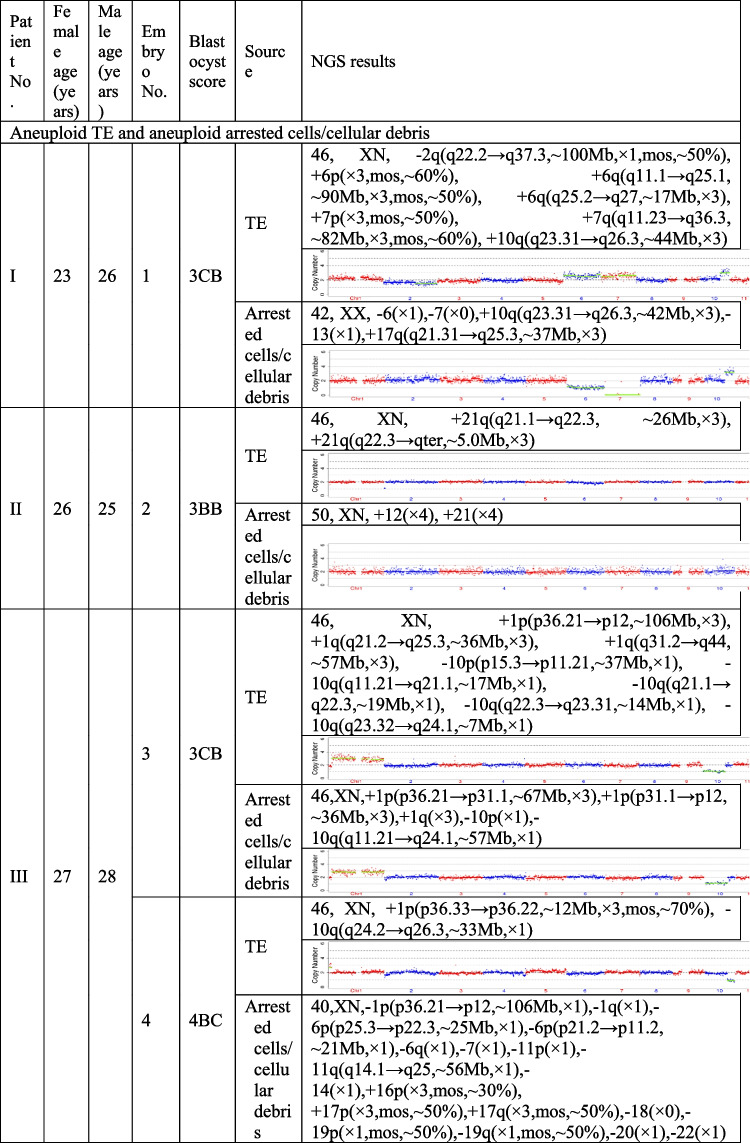

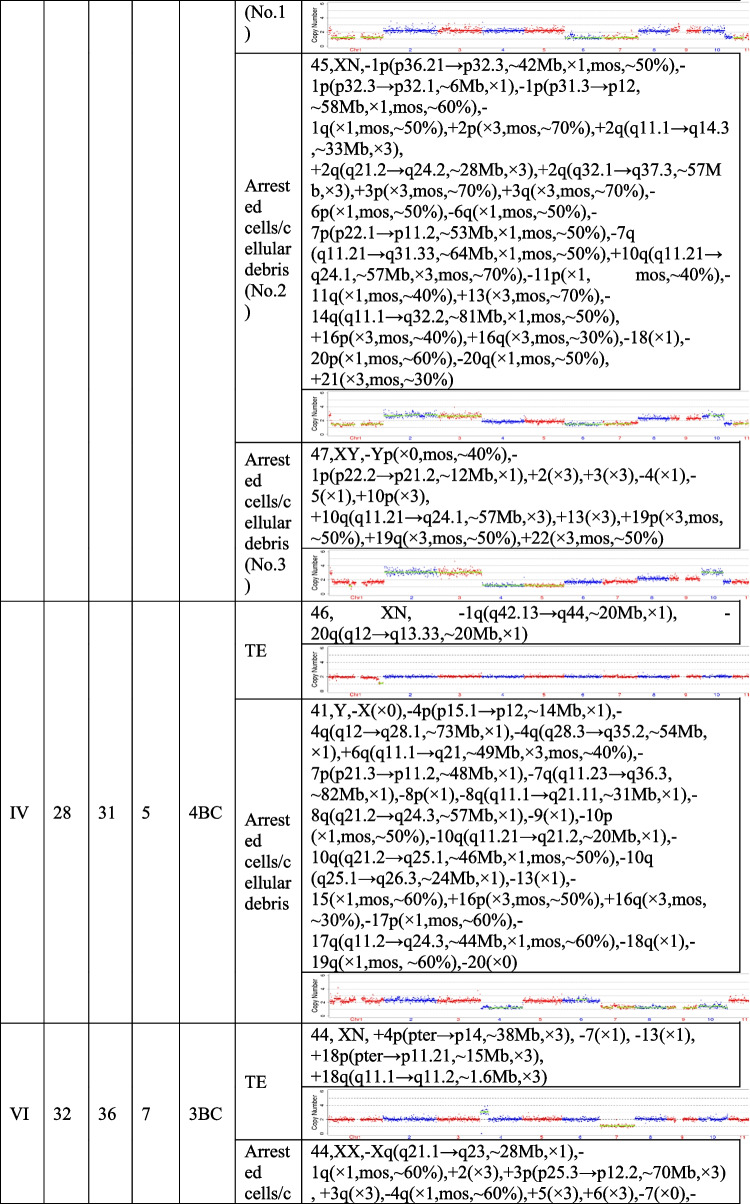

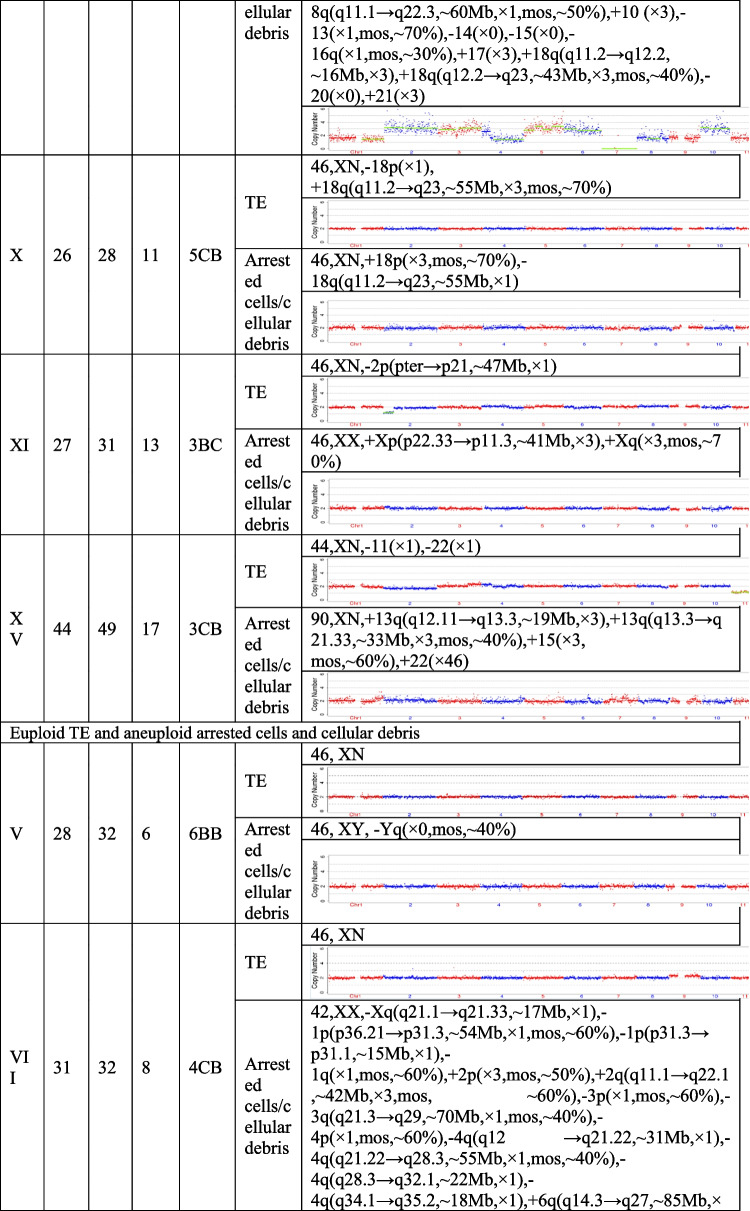

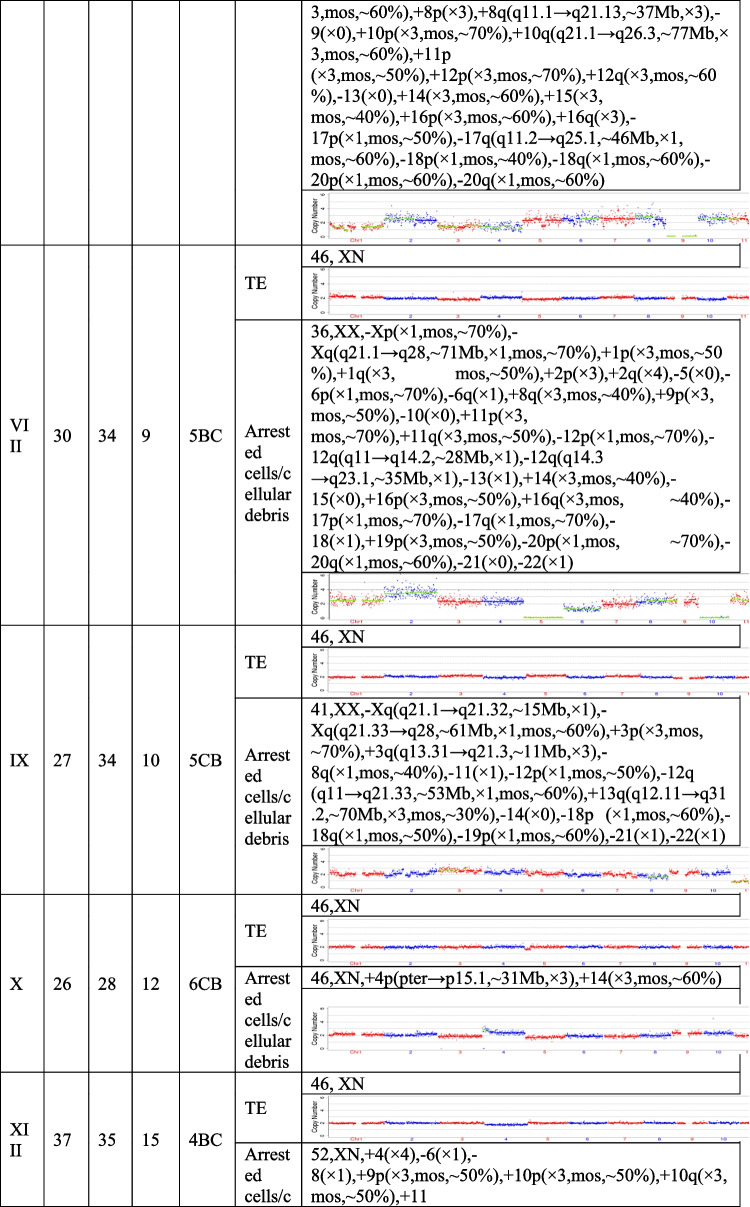

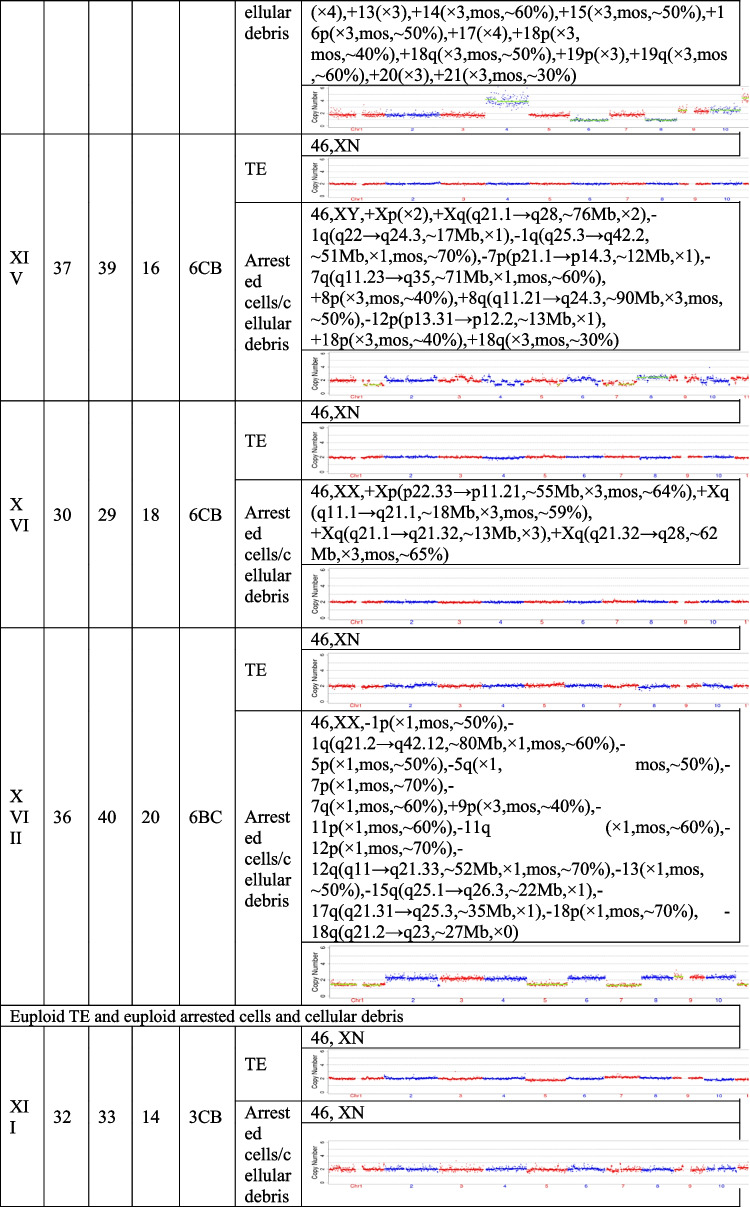

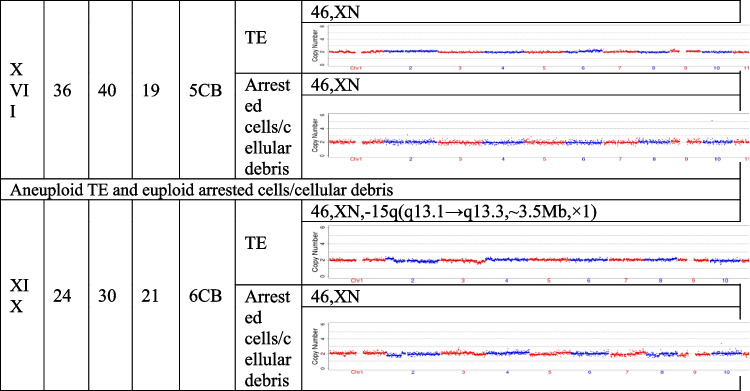
The clinical information and NGS results of the TE biopsies and the corresponding arrested cells/cellular debris

TE, trophectoderm; NGS, next-generation sequencing; XN = XY or XX.

The TE biopsy results revealed that 47.6% (10/21) of blastocysts were aneuploidies or mosaicism and 52.4% (11/21) of blastocysts were euploidies. In the ten aneuploid blastocysts, nine corresponding arrested cells/cellular debris were abnormal, including seven presenting additional chromosomal rearrangements or increased abnormal chromosomal fragments (No. 1, 2, 4, 5, 7, 13, and 17). Interestingly, the arrested cells/cellular debris of the other one presented euploidy (No. 21). In the 11 euploid blastocysts, nine corresponding arrested cells/cellular debris (81.8%) were aneuploid fragments (No. 6, 8, 9, 10, 12, 15, 16, 18, and 20), and only two pairs of TE biopsies and arrested cells/cellular debris (No. 14 and 19) were found to be both euploid. In total, 18 groups of arrested cells/cellular debris (85.7%) removed from the blastocysts were abnormal.

**Fig. 2:**
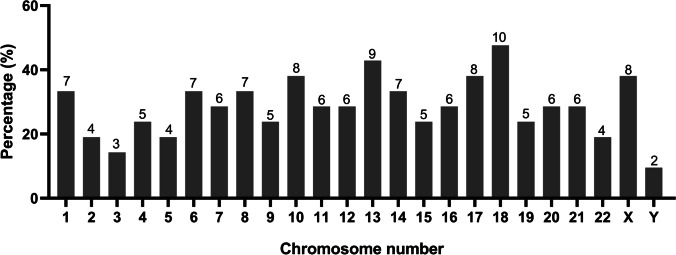
Percentage of affected chromosomes in the 21 groups of arrested cells/cellular debris

In addition, we calculated the abnormal chromosomal fragments in the 21 groups of arrested cells/cellular debris (the three arrested cells/cell debris in the No. 4 sample were considered as a whole). All the chromosomes were affected. And the abnormal proportion of chromosome 18 was 57.6% (10/21), that of chromosome 13 accounted for 42.9% (9/21), and 38.1% for chromosomes 10, 17 and X (8/21) (Fig. 2).

## Discussion

The present study examined both TE cells and arrested cells/cellular debris expelled from the selected blastocysts. Also, 47.6% (10/21) of the detected blastocysts were aneuploidies or mosaicism. And 18 pairs out of the 21 blastocyst-arrested cells/cellular debris (85.7%) were expelled from the blastocysts with additional chromosomal rearrangements and increased abnormal chromosomal fragments. Among them, nine aneuploid blastocysts and nine euploid blastocysts expelled aneuploid arrested cells/cellular debris. The results confirmed that aneuploid and mosaic embryos were common phenomena during the early embryonic development, and supported the hypothesis that the elimination of chromosomally abnormal cells during early embryonic development might be a self-correction process, allowing to minimize the proportion of abnormal cells in subsequent embryos.

A majority of studies have reported that aneuploidies and mosaic embryos are prevalent during early embryonic development. Munne et al. found that 56% of embryos with the best morphology and development belonged to aneuploidies in patients younger than 35 by detecting more than 6000 embryos, and the proportion was even higher in patients 41 and older [6]. Mertzanidou et al. demonstrated that 71.4% (10/14) of good-quality embryos were mosaic [19]. Our studies examined 21 blastocysts by TE biopsies, and the proportion of aneuploidies and mosaicism was 47.6% (10/21), confirming the physiological phenomenon again.

PGT-A is applied in IVF/ICSI cycles to select euploid embryos, aiming to improve pregnancy outcomes. However, recent studies showed that PGT-A seemed to have no beneficial effects in good-prognosis patients [26, 27]. This is because mosaicism is a physiological feature of early human embryos and had the ability to develop a viable pregnancy [10–12]. Previous evidence also suggests that initial mosaicism or aneuploidy within the embryo may be self-limiting by demonstrating a decrease in the incidence of mosaicism or aneuploidy in vitro over time [13, 15]. However, the underlying mechanisms of normalization of aneuploid cells in human embryos throughout development, including abnormal cell elimination and embryonic self-correction, are confined to a theoretical basis.

Animal studies have suggested that self-correction process involves in cell arrest and apoptosis. Bolton et al. revealed that aneuploid cells in the fetal lineage and placental lineage had different correction ways by using a mouse model of embryo mosaicism caused by a spindle assembly checkpoint inhibitor during the four- to eight-cell division [8]. Aneuploid cells in the fetal lineage were eliminated by apoptosis; nevertheless, those in the placental lineage were eliminated by proliferative defects. Subsequently, their team further showed that aneuploid cells in the fetal lineage were dominantly eliminated in a p53-dependent process involving both autophagy and apoptosis before, during, and after implantation [28]. And mosaic mouse embryos increased cellular proliferation to compensate cell death during implantation stages [28]. Moreover, Daughtry et al. discovered that in rhesus monkey embryos, part of or all abnormal chromosomes as a form of fragments within micronuclei could be lost during cell division, whereas abnormal blastomeres with extensive DNA damage do not progress further into blastocysts [29]. Transcriptomic analyses also have identified that novel candidate genes related to apoptosis regulate early embryo survival and death [30].

In our present study, the comparison of NGS results between TE biopsies and the corresponding arrested cells/cellular debris showed that a total of 18 embryos expelled aneuploid arrested cells/cellular debris, including nine aneuploid blastocysts and nine euploid blastocysts. Especially, seven arrested cells/cellular debris from the aneuploid blastocysts presented additional chromosomal rearrangements or increased abnormal chromosomal fragments. The results directly exhibited strong evidence of the human embryonic self-correction ability. Because of the limited experiment samples, we did not conduct further experiments to investigate mechanism occurred in the process. But according to the animal studies, we can speculate that the arrested cells/cellular debris may be expelled from embryos by cell arrest and apoptosis. In addition, the phenomenon that two euploidies and one aneuploidy expelled euploid arrested cells/cellular debris indicates that the self-correction mechanism in the embryos can be overwhelming sometimes, resulting in expelling some euploid cells. Furthermore, due to the congruency between TE biopsies and arrested cells/cellular debris, these arrested cells/cellular debris are not a suitable source for PGT-A.

Orvieto et al. and Lagalla et al. had analyzed arrested cells/cell debris before [31, 32]. Orvieto et al. called these residues in the zona pellucida as cell debris/fragments and compared them with the corresponding embryos to prove that human embryos had the ability to self- correction. They also mentioned that because of the self-correction mechanism, the efficacy of noninvasive PGT-A (niPGT-A) which analyzed the cell-free DNA in the spent culture media need be suspected. We are in favor of the viewpoint as well. As to Lagalla et al., they analyzed the difference between these excluded cells and TE biopsies and providing the evidence for a potential mechanism of “aneuploidy rescue” of the embryo. However, Orvieto et al.’s research used day 3 discarded human embryos undergoing PGT-M which further cultured until days 5–6. And Lagalla et al. only analyzed excluded cells from irregularly cleaved embryos, not including excluded cells from normally cleaved embryos. Our study used PGT-embryo samples, including normal and abnormal blastocysts. The results may more represent and give evidence for the physiological self-correction phenomenon. Besides, they used array comparative genomic hybridization (aCGH), but our study used NGS technology. As we all know, NGS shows better application than aCGH, with superior sensitivity and higher precision [33]. Furthermore, Orvieto et al.’s study only included 11 pairs of blastocytes and arrested cells/cellular debris, and Lagalla et al. only included 12 pairs. Although it was still small, the sample size of our study was almost twice than theirs to further supplement data in the aspect.

Some inevitable limitations need to be acknowledged and discussed here. First, for ethical reasons, we did not separate ICM and TE for sequencing. Therefore, the source of arrested cells/cellular debris could not be confirmed. Second, it is an observational study, and no experiment data were provided to prove mechanisms because of the limited samples. In the future, studies with a large size are required. The arrested cells/cellular debris samples, ICM and TE biopsies can be separately collected for RNA sequencing to investigate potential self-correction mechanisms of human embryos.

## Conclusion

Both mosaic and aneuploid embryos are common during early embryonic development. The residual arrested cells/cellular debris in the zona pellucida of blastocysts provide evidence of self-correction mechanism during early embryonic development.

## Data Availability

The datasets generated during and/or analyzed during the current study are available from the corresponding author on reasonable request.
